# Randomized Controlled Trial of a Mailed Toolkit to Increase Use of Body Mass Index Percentiles to Screen for Childhood Obesity

**Published:** 2009-09-15

**Authors:** Barbara A. Dennison, Joseph Nicholas, Rachel de Long, Megan Prokorym, Ian Brissette

**Affiliations:** Dr Dennison is affiliated with the New York State Department of Health and State University of New York at Albany, Albany, New York; University of Rochester School of Medicine, Rochester, New York. At the time of this study, Dr Nicholas was affiliated with the State University of New York at Albany, Albany, New York; New York State Department of Health and State University of New York at Albany, Albany, New York; New York State Department of Health, Albany, New York; New York State Department of Health, Albany, New York

## Abstract

**Introduction:**

Despite epidemic increases in childhood obesity rates, many providers fail to diagnose obesity. Body mass index (BMI)-for-age percentiles are the recommended screening test. We evaluated whether mailing a toolkit to physicians would increase use of sex-specific BMI-for-age percentiles to screen for childhood obesity.

**Methods:**

We assigned a random sample of family physicians and pediatricians from New York State’s medical licensing database to either intervention or control groups in the summer of 2004. At baseline and at follow-up, we sent physicians a survey that asked how often they used various screening methods to identify childhood obesity. Between the surveys, we sent physicians in the intervention group a toolkit that consisted of professional guidelines for childhood obesity screening, a tool for calculating BMI, BMI-for-age growth charts, and educational information.

**Results:**

At follow-up, more physicians in the intervention group than in the control group reported using BMI percentiles to screen for childhood obesity. Compared with physicians in the control group, physicians in the intervention group had a larger increase in their routine use of BMI percentiles to screen children aged 2 to 5, 6 to 11, and 12 to 20 years, although the differences in the older 2 groups did not attain statistical significance.

**Conclusion:**

Directly mailing an educational toolkit to physicians can have a small but positive effect on clinical practice.

## Introduction

Childhood obesity is a national health problem (1), but the public health and medical communities have struggled to implement screening strategies among children and adolescents. Sex-specific body mass index (BMI)-for-age percentiles are the preferred method for obesity screening in youths aged 2 to 20 years (2). BMI percentiles correlate closely with standard measurements of adiposity (3,4); track into adulthood, thereby predicting adult obesity (5-7); and can be reliably measured in the office setting. BMI percentiles appear to more accurately assess weight status than do traditional assessments (8).

Sex-specific BMI-for-age growth charts are available, but few providers have implemented these standards. A national survey of pediatric providers in 2002 showed that only 12% of pediatricians routinely use BMI percentiles to assess weight status (9); a 2002 North Carolina survey found that only 11% always use BMI percentiles (8). BMI is not routinely documented in patients' charts (10), childhood overweight and obesity are dramatically underrecognized (11-13), and nondiagnosed children are less likely to receive dietary counseling, exercise counseling, screening tests for comorbid conditions, or other interventions (8). BMI percentiles are used even less to screen for obesity in young children (11).

Public health policy and clinical medicine intersect in the development and adoption of clinical practice guidelines. Lack of adherence to clinical practice guidelines is well established (14-16). Barriers to guideline adherence include awareness, familiarity, self-efficacy, and practice inertia (14). Although provider education is commonly used to promote the use of specific guidelines, additional strategies are often necessary (17).

In 2003, the New York State Department of Health developed a toolkit for providers to encourage them to use sex-specific BMI-for-age percentiles. The primary goals of this toolkit were to reemphasize the dangers of childhood obesity and distribute to providers the educational and practice resources needed to adopt obesity screening based on BMI percentiles. Although toolkits can improve clinical preventive practices (18,19), no evidence supports the effectiveness of mailing them to providers.

We assessed physicians' use of BMI-for-age percentiles to screen for childhood obesity and whether mailing the toolkit to pediatric and family practice physicians affected their use of BMI percentiles. A secondary goal was to measure physicians' preferences for further education and training in diagnosing and managing obesity among children.

## Methods

### Sample

**Figure 1:**
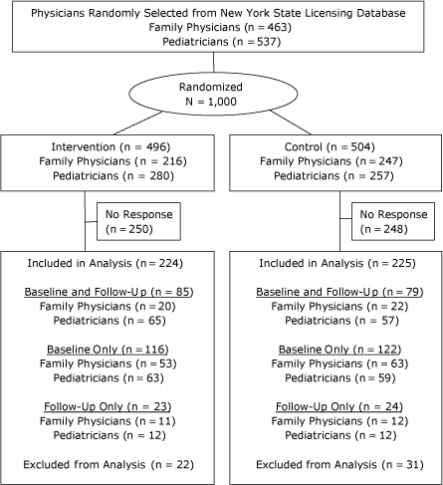
Selection, randomization, and inclusion of physicians licensed in New York State for survey on using body mass index-for-age percentiles to screen for childhood obesity.

The target population for this study was pediatricians and family physicians licensed in New York State to provide primary health care for youths aged 2 to 20 years. We obtained a random sample of physicians who reported their primary practice as either pediatrics or family practice from the state department of health's medical licensing database and randomly assigned them to either the intervention (n = 496) or control group (n = 504) (Figure 1). Physicians who reported on either the baseline or follow-up survey that they were retired or did not see pediatric patients for primary care were excluded from analysis.

### Surveys and intervention

In September 2004, we mailed (via the US Postal Service) surveys to the 1,000 randomly selected physicians. Surveys asked how frequently physicians used sex-specific BMI-for-age percentiles to assess "excess weight" (ie, screen for childhood obesity) for children in each of the following age groups: 2 to 5, 6 to 11, and 12 to 20 years. Surveys also asked about frequency of using BMI, weight-for-age percentile, percentage ideal body weight, weight-for-height percentile, and crossing percentiles. Frequency was reported on a 5-point Likert scale (4 = most of the time, 3 = often, 2 = sometimes, 1= rarely, 0 = never). For some analyses, the responses "most of the time" and "often" were collapsed to define "routine practice," and the other responses were collapsed to define practices not routinely performed. We also collected the following information about each practice: staff responsible for measuring height and weight, staff responsible for plotting measurements on growth charts, primary practice structure (eg, private, hospital-based, community health center), geographic location (rural, urban, suburban), and percentage of privately insured patients. The survey also asked for physicians' specialty (family physician, pediatrics, internal medicine/pediatrics, general practice), year postgraduate medical training was completed, and whether they supervise medical students or residents. To improve response rates, baseline surveys were mailed a second time to physicians who did not respond. Surveys were returned by mail in an addressed, postage-paid return envelope, which we included with the survey.

In December 2004, we mailed a toolkit to the 496 physicians in the intervention group. The toolkit promoted the use of BMI-for-age percentiles to screen youths aged 2 to 20 years for obesity and consisted of the following:

- BMI calculator
- sex-specific BMI-for-age percentile growth charts
- laminated office chart summarizing steps to calculate, plot, and interpret BMI
- printed recommendations of the American Academy of Pediatrics to prevent pediatric overweight (20)
- additional professional resources, including growth chart information, links to training modules (21), and links to the Bright Futures in Practice (22), a collection of patient and family questionnaires on nutrition

We also included a letter highlighting the BMI percentiles-based screening recommendations and the purpose of the mailing, signed by the New York State commissioner of health, the president of the New York State chapter of the American Academy of Pediatrics (District II), and the president of the New York State Academy of Family Physicians.

In April 2005, we mailed follow-up surveys that repeated the question from the baseline survey on frequency of use of BMI-for-age percentiles to assess weight status. The follow-up survey also ascertained whether physicians had received additional information related to childhood obesity since the first survey and any perceived needs for training to improve screening for and treatment of childhood obesity. The study design, study protocol, and all data collection, management, and publication strategies were approved by the institutional review board of the New York State Department of Health. Participants gave written consent to participate as part of both the baseline and follow-up surveys.

### Statistical analyses

Data were analyzed by using SAS version 8.2 (SAS Institute, Inc, Cary, North Carolina). We determined comparability of the control and intervention groups by using χ^2^ tests for categorical variables, nonparametric Wilcoxon rank-sum tests for ordered variables, and Student *t* tests for continuous variables. All statistical tests were 2-sided, but directional (1-sided) *t* tests were used to evaluate the primary study hypothesis that frequency of using BMI-for-age percentiles to screen for obesity would increase between baseline and follow-up among providers in the intervention group compared with the control group. To test the difference in routine use of BMI-for-age percentiles between physicians who completed their training after 1998 (when expert committee recommendations were released [2]) and those who completed their training in 1998 or earlier, we combined data on the reported frequency of routine use across 3 patient age categories (2-5, 6-11, and 12-20 years) and evaluated them by using χ^2^ tests. To identify which physician characteristics independently predicted routine use of BMI-for-age percentiles in the baseline survey, we ran separate multivariate logistic regression models for each of the 3 age groups. Differences were considered significant at *P* < .05.

## Results

A total of 402 physicians returned baseline surveys (response rate, 40%). Most were men and practiced in either an urban or suburban setting. The control and intervention groups did not differ on any measured variables (Table 1).

A total of 211 physicians returned follow-up surveys (response rate, 21%) (Figure 1). We excluded from analysis 53 physicians who reported on either the baseline or follow-up survey that they were retired or did not see pediatric patients for primary care. We used matched follow-up and baseline questionnaire data (n = 164) to evaluate changes over time in physician-specific practice behavior. Pediatricians were more likely than family physicians to return both surveys; therefore, they are overrepresented in the sample used to evaluate changes in practice behavior. No other physician characteristic measured at baseline (Table 1) was significantly associated with participating in both the baseline and follow-up surveys.

### Pediatric practice in New York State

Physicians reported that either medical assistants (35%) or licensed practical nurses (27%) were the staff in their practice who most often measured children's height and weight. In contrast, most physicians (55%) reported that physicians were the staff who most often plotted height and weight on growth charts.

At baseline, the reported use of BMI percentiles significantly differed by physician specialty, year medical training was completed, and practice setting (Table 2). Pediatricians used BMI-for-age percentiles to screen for childhood obesity more often than did family physicians. More physicians who completed their medical training after 1998, when the expert committee recommendations were released (2), routinely used BMI percentiles than did those who completed their training in 1998 or earlier (45% vs 32%, respectively; χ^2^ = 14.19, *P* < .01). Physicians who practiced in an urban setting also used BMI percentiles more often than did those who practiced in rural or suburban settings.

Multivariate analyses indicated practice setting, year of completing training, and specialty independently predicted routine use of BMI percentiles (Table 3). Although practicing in an urban setting was associated with increased use of BMI percentiles for all 3 age groups, completing medical training after 1998 was associated with increased use only for children aged 6 to 20 years, and specializing in pediatrics was associated with increased use only for children aged 6 to 11 years.

### Effect of toolkit

**Figure 2:**
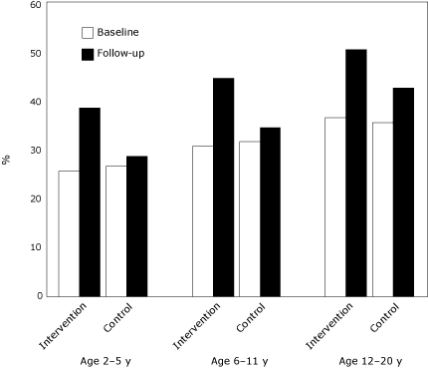
Percentage of New York State physicians who reported routine (always or most of the time) use of body mass index-for-age percentiles to screen for childhood obesity, by children's age group

Between baseline and follow-up, the intervention group increased its use of BMI percentiles across the 3 age groups (Table 4). Compared with the increase in the control group, the increase in the intervention group was significant for children aged 2 to 5 years and approached significance for children aged 6 to 20 years. Physicians in the intervention group increased their routine use of BMI percentiles by 50%, 45%, and 38%, respectively, for the 3 age groups studied (Figure 2). Most physicians in the intervention group reported that the toolkit was either somewhat or very helpful (72%) and that it influenced the ways they assess weight status of their pediatric patients (67%).

**Table:** 

**Age of Child, y**	**Baseline Survey**		**Follow-Up Survey**	
	**Intervention, %**	**Control, %**	**Intervention, %**	**Control, %**
2-5	26	27	39	29
6-11	31	32	45	35
12-20	37	36	51	43

During the study period, a number of other public and private efforts to address childhood obesity took place. At follow-up, more than half the physicians reported that they had received information during the past 3 months from sources other than the New York State Department of Health on diagnosing or treating overweight and obesity among children. There was no significant difference between the study groups in reported receipt of information from sources other than the New York State Department of Health. The most common sources of information were professional medical organizations. Among physicians in the control group who reported receiving such materials, 28% reported that the materials influenced the way they assessed pediatric weight status.

### Physician preferences for further training

Physicians reported a high level of interest in training to better manage obesity in children, with specific interests in behavior management strategies (44%), modification of dietary practices (48%), and physical activity patterns (43%). Forty-eight percent were interested in guidance on parenting techniques, while 34% were interested in how to address family conflicts or concerns. Sixty-nine percent selected professional guidelines as the tool they would most prefer to improve their ability to treat overweight children, followed by BMI growth charts (57%) and continuing medical education courses at local meetings (55%).

## Discussion

This study found that mailing an educational toolkit to physicians can affect their self-reported practice patterns. To our knowledge, this is the only randomized controlled trial that evaluated a public health intervention to increase physicians' compliance with recommended screening strategies for childhood obesity. The study design allows the effect of the toolkit to be distinguished from temporal changes and other confounding influences resulting from increased public, professional, and commercial attention to pediatric obesity and the use of BMI percentiles for screening. The random selection of participants from the medical licensing registry provides a population-based sample, representative of New York State's primary care pediatricians and family physicians. By including family practice physicians in the sampling frame, the diversity of pediatric care providers is also more representative than in previous studies (8,9,12,13).

Additional strengths of this study include the high physician response rate for the baseline survey, which was twice that reported in a similar study of physicians (40% vs 19%) (9), and the collection of individual-level data for matched analysis. The baseline frequency of BMI percentiles-based obesity screening in this study is also consistent with previously published rates by pediatric care providers in North Carolina (8) and nationwide (9,23).

In the 9 years since expert committee recommendations were published, routine use of BMI-for-age percentile-based screening increased from 12% (9) to 30% (for 2- to 5-year-old children) to 40% (for 12- to 20-year-old adolescents) among physicians who returned the baseline survey (n = 400) in this study. Although physicians who completed their training after the recommendations were published were more likely to routinely use BMI percentiles, overall adoption has been slow. Lack of awareness of guidelines has been repeatedly documented; a 2001 survey found that fewer than 20% of pediatricians and family physicians surveyed nationwide were aware of the committee recommendations (23), and similar levels of awareness were reported in 2004 among Massachusetts physicians (24). Professional guidelines were the preferred educational tool for physicians in our sample.

Several limitations to our study exist. No information is available for nonresponders, and we cannot quantify the magnitude of response bias present in these findings. The accuracy of physicians' self-report is also uncertain; we may have measured provider intentions rather than actual practices in some instances. Surveys were mailed to individual providers, and we were unable to determine if surveys were distributed to more than 1 physician in a practice. Practices may have standardized procedures for assessing weight status or screening for childhood obesity, which could influence physician behavior. Bias may have been introduced if a practice decided to adopt the use of BMI percentiles after the toolkit mailing and more than 1 physician in the practice was surveyed.

Our sample size was small; we could confirm increased use of BMI percentiles only for the youngest age group of patients. A larger sample might have demonstrated statistical significance for the older age groups. Because follow-up was done only 5 months after the intervention, our findings may underestimate the effect of the toolkit on provider practice.

This study did not collect information about barriers that exist for physicians in implementing BMI percentile-based obesity screening. In addition to traditional barriers of knowledge and cost, physicians may be skeptical about their ability to manage or treat pediatric obesity (25), which may diminish their motivation to detect it. Previously identified barriers to obesity treatment include patient and parental resistance to change, lack of time and reimbursement, and lack of effective treatments (9,24,26).

Our findings of a positive effect on guideline adherence are in contrast to a recent, systematic review of the medical literature that found little evidence that printed educational materials have a meaningful effect (27). "Dissemination only" techniques have been described as unlikely to change practice dramatically (24,28). Mailed, unsolicited materials have been categorized as weak interventions at best (29). Personal and individualized interventions, such as academic detailing and peer audit and feedback, appear to be more effective in changing practice patterns (29); these efforts, however, are substantially more time- and labor-intensive. Our intervention coincided with broad societal concern over obesity and provided specific practice tools in addition to guidelines; both factors may have contributed to the benefit seen here. Receiving consistent messages about the use of BMI percentiles for childhood obesity screening from other sources may have made physicians more attentive to the toolkit and responsive to its messages (30).

The limitations illustrate some of the practical challenges in integrating rigorous research in public health practice. Lower than optimal response rates, presence of competing interventions and resources, and the need to balance evaluation design with the broader goals and implementation timeframes of population-based interventions are typical realities of this type of research. Such inherent limitations most likely contribute to the paucity of published evidence on the effectiveness of routine public health educational interventions such as this one.

Our findings suggest several areas for further research. A better understanding is needed of the barriers and reasons for resistance to the use of BMI percentiles for childhood obesity screening. Our sample reported that multiple levels of practice staff are responsible for pediatric growth monitoring, which indicates that getting practices to adopt and routinely use BMI percentiles may require a systemic change in the practice setting. The public health effect of this study is also uncertain. Although the early and accurate identification of childhood obesity may increase dietary and exercise counseling (11,24), early diagnosis of childhood obesity may not improve long-term health outcomes.

In conclusion, we found that directly mailing an educational toolkit to physicians, in the absence of detailing or other facilitative methods, can have a small but positive effect on clinical practice. This finding can be useful to public health agencies because distributing materials in this way is easier and cheaper than more intensive educational methods.

## Figures and Tables

**Table 1:** Characteristics of 402 Physicians Who Returned a Survey on Using BMI-for-Age Percentiles to Screen for Childhood Obesity, New York State, September 2004

**Characteristic**	Intervention, n (%)^a^ (n = 201)	Control, n (%)^a^ (n = 201)	*P* Value^b^
**Sex**			
Male	107 (53)	109 (54)	.91
Female	81 (40)	81 (40)	
Missing data	13 (7)	11 (6)	
**Year medical training completed**			
Median	1989	1990	.93
Range	1943-2004	1943-2004	
**Specialty**			
General pediatrics	122 (61)	112 (56)	.56
Family medicine	64 (32)	79 (39)	
Internal medicine/pediatrics	6 (3)	4 (2)	
General practice	3 (2)	2 (1)	
Missing data	6 (3)	4 (2)	
**Practice setting**			
Rural	27 (13)	36 (18)	.23
Urban	97 (48)	81 (40)	
Suburban	64 (32)	72 (36)	
Other	0	2 (1)	
Missing data	13 (7)	10 (5)	
**Practice structure**			
Private, solo	50 (25)	51 (25)	.86
Private, group	86 (43)	78 (39)	
Managed care organization	10 (5)	9 (5)	
University-based	16 (8)	19 (10)	
Free-standing clinic	11 (6)	11 (6)	
Hospital-based	12 (6)	16 (8)	
Other	3 (2)	7 (4)	
Missing data	13 (7)	10 (5)	
**Supervise medical students/residents**			
Yes	116 (58)	115 (57)	.88
No	73 (36)	76 (38)	
Missing	12 (6)	10 (5)	
**% of patients who are privately insured**			
0	11 (6)	4 (2)	.21
<25	49 (24)	61 (30)	
25-50	29 (14)	35 (17)	
>50	87 (43)	81 (40)	
Missing data	25 (12)	20 (10)	

Abbreviation: BMI, body mass index.

a All values are n (%) except year medical training completed. Percentages may not total 100 because of rounding.

b Nonparametric Wilcoxon rank-sum test for year medical training completed; all other comparisons use χ^2^ tests.

**Table 2:** Use of BMI-for-Age Percentiles to Screen for Childhood Obesity, by Age of Children, New York State, September 2004

Physician Characteristic	Age of Children, y					

	2-5		6-12		12-20	

	n^a^	Mean (SD) Score^b^	n^a^	Mean (SD) Score^b^	n^a^	Mean (SD) Score^b^
**Total sample**	362	1.7 (1.5)	365	1.9 (1.5)	364	2.0 (1.5)
**Sex**						
Female	148	1.8 (1.5)	149	2.0 (1.5)	150	2.2 (1.5)
Male	198	1.6 (1.5)	199	1.7 (1.5)	196	1.9 (1.5)
* P* value^c^		.29		.06		.16
**Medical training completed**						
1998 and earlier	282	1.6 (1.5)	285	1.8 (1.5)	285	2.0 (1.5)
After 1998	80	2.0 (1.7)	80	2.3 (1.4)	79	2.5 (1.4)
* P* value^c^		.05		.003		.005
**Practice setting**						
Rural	57	1.2 (1.3)	57	1.3 (1.3)	58	1.4 (1.3)
Urban	162	2.0 (1.5)	161	2.3 (1.5)	161	2.4 (1.5)
Suburban	124	1.5 (1.4)	126	1.6 (1.4)	124	1.8 (1.5)
* P* value^d^		.001		.001		.001
**Specialty**						
Family medicine	126	1.8 (1.5)	128	1.5 (1.4)	128	1.8 (1.5)
Pediatrics	214	2.2 (1.4)	215	2.0 (1.5)	217	2.2 (1.5)
* P* value^c^		.08		.002		.02
**Supervise medical students/residents**						
Yes	139	1.8 (1.5)	140	2.0 (1.5)	137	2.2 (1.5)
No	208	1.6 (1.5)	210	1.8 (1.5)	211	2.0 (1.5)
* P* value^c^		.20		.15		.26

Abbreviation: BMI, body mass index.

a Number of physicians who answered a particular question.

b Mean Likert score (5-point scale): 4 = most of the time, 3 = often, 2 = sometimes, 1 = rarely, and 0 = never.

c *P* value 2-sided; Student *t* tests for mean Likert score.

d *P* value based on *F* statistic from analysis of variance for mean Likert score.

**Table 3:** Multivariate Logistic Regression Models for Routine Use of BMI-for-Age Percentiles to Screen for Childhood Obesity, New York State, September 2004

Variable	Age of Children, y									

	2-5		6-11		12-20	

	OR (95% CI)	*P* Value	OR (95% CI)	*P* Value	OR (95% CI)	*P* Value
**Sex**										
Female	1 [Reference]		1 [Reference]		1 [Reference]	
Male	1.02 (0.61-1.70)	.94	0.81 (0.49-1.33)	.40	0.84 (0.52-1.37)	.50
**Practice setting**										
Rural	1 [Reference]	1 [Reference]	1 [Reference]
Urban	3.11 (1.36-7.14)	.007	3.18 (1.43-7.04)	.004	4.06 (1.92-8.58)	<.001
Suburban	1.83 (0.78-4.28)	.16	1.50 (0.66-3.42)	.33	1.72 (0.80-3.70)	.17
**Specialty**										
Family medicine	1 [Reference]	1 [Reference]	1 [Reference]
Pediatrics	1.05 (0.60-1.83)	.87	1.77 (1.01-3.11)	.047	1.25 (0.74-2.12)	.40
**Year medical training completed**										
In or before 1998	1 [Reference]	1 [Reference]	1 [Reference]
After 1998	1.55 (0.88-2.71)	.13	2.07 (1.18-3.62)	.01	1.96 (1.34-3.40)	.02
**Supervise medical students/residents**										
Yes	1 [Reference]	1 [Reference]	1 [Reference]
No	0.97 (0.58-1.62)	.91	0.80 (0.48-1.32)	.38	0.87 (0.54-1.41)	.57

Abbreviations: BMI, body mass index; OR, odds ratio; CI, confidence interval.

**Table 4:** Change in Frequency of Using BMI-for-Age Percentiles to Screen for Childhood Obesity in Children Among Intervention and Control Physicians, New York State, April 2005

Age of Children, y	Intervention Group			Control Group				Difference in Change^b^
	Baseline	Follow-Up	Change^a^	Baseline	Follow-Up	Change^a^		
**2-5**								
No. of participants	80	84	77	72	76		67	144
Mean (SE)^c^	2.72 (0.16)	3.13 (0.16)	0.47 (0.13)	2.79 (0.18)	2.79 (0.17)		0.07 (0.16)	0.40 (0.21)
* P* value^d^			<001				.33	.03
**6-11**								
No. of participants	80	84	76	73	71	63		139
Mean (SE)^c^	2.95 (0.15)	3.33 (0.16)	0.46 (0.12)	2.95 (0.18)	3.00 (0.18)	0.13 (0.16)		0.33 (0.20)
* P* value^d^			<.001			.21		.07
**12-20**								
No. of participants	79	84	75	72	72		63	138
Mean (SE)^c^	3.10 (0.16)	3.46 (0.16)	0.45 (0.13)	2.99 (0.19)	3.12 (0.19)		0.13 (0.18)	0.32 (0.21)
* P* value^d^			<.001				.24	.08

Abbreviation: BMI, body mass index; SE, standard error.

a Change (follow-up minus baseline) calculated for physicians with both baseline and follow-up data by using item-wise deletion for missing data. The number of study participants reflects the number of physicians with data for an item at both baseline and follow-up.

b Change in intervention group mean minus change in control group mean.

c Mean Likert score (5-5-point scale): 4 = most of the time, 3 = often, 2 = sometimes, 1 = rarely, and 0 = never.

d *P* values are 1-sided, paired Student *t* tests for change (follow-up minus baseline) or difference in change (between intervention group and control group).

## References

[1] (2001). The Surgeon General's call to action to prevent and decrease overweight and obesity, 2001.

[2] Barlow SE, Dietz WH (1998). Obesity evaluation and treatment: expert committee recommendations. Pediatrics.

[3] Mei Z, Grummer-Strawn LM, Pietrobelli A, Goulding A, Goran MI, Dietz WH (2002). Validity of body mass index compared with other body-composition screening indexes for the assessment of body fatness in children and adolescents. Am J Clin Nutr.

[4] Pietrobelli A, Faith MS, Allison DB, Gallagher D, Chiumello G, Heymsfield SB (1998). Body mass index as a measure of adiposity among children and adolescents: a validation study. J Pediatr.

[5] Freedman DS, Khan LK, Serdula MK, Dietz WH, Srinivasan SR, Berenson GS (2005). The relation of childhood BMI to adult adiposity: the Bogalusa Heart Study. Pediatrics.

[6] Guo SS, Chumlea WC (1999). Tracking of body mass index in children in relation to overweight in adulthood. Am J Clin Nutr.

[7] Guo SS, Wu W, Chumlea WC, Roche AF (2002). Predicting overweight and obesity in adulthood from body mass index values in childhood and adolescence. Am J Clin Nutr.

[8] Perrin EM, Flower KB, Ammerman AS (2004). Body mass index charts: useful yet underused. J Pediatr.

[9] Barlow SE, Dietz WH, Klish WJ, Trowbridge FL (2002). Medical evaluation of overweight children and adolescents: reports from pediatricians, pediatric nurse practitioners, and registered dietitians. Pediatrics.

[10] Dorsey KB, Wells C, Krumholz HM, Concato JC (2005). Diagnosis, evaluation, and treatment of childhood obesity in pediatric practice. Arch Pediatr Adolesc Med.

[11] Cook S, Weitzman M, Auinger P, Barlow SE (2005). Screening and counseling associated with obesity diagnosis in a national survey of ambulatory pediatric visits. Pediatrics.

[12] O'Brien SH, Holubkov R, Reis EC (2004). Identification, evaluation, and management of obesity in an academic primary care center. Pediatrics.

[13] Louthan MV, Lafferty-Oza MJ, Smith ER, Hornung CA, Franco S, Theriot JA (2005). Diagnosis and treatment frequency for overweight children and adolescents at well child visits. Clin Pediatr (Phila).

[14] Cabana MD, Rand CS, Powe NR, Wu AW, Wilson MH, Abboud PC (1999). Why don't physicians follow clinical practice guidelines?: a framework for improvement. JAMA.

[15] Leape LL, Weissman JS, Schneider EC, Piana RN, Gatsonis C, Epstein AM (2003). Adherence to practice guidelines: the role of specialty society guidelines. Am Heart J.

[16] Brindis RG, Sennett C (2003). Physician adherence to clinical practice guidelines: does it really matter?. Am Heart J.

[17] Lomas J, Anderson GM, Domnick-Pierre K, Vayda E, Enkin MW, Hannah WJ (1989). Do practice guidelines guide practice? The effect of a consensus statement on the practice of physicians. N Engl J Med.

[18] Hogg W, Huston P, Martin C, Saginur R, Newbury A, Vilis E (2006). Promoting best practices for control of respiratory infections: collaboration between primary care and public health services. Can Fam Physician.

[19] Melnikow J, Kohatsu ND, Chan BK (2000). Put prevention into practice: a controlled evaluation. Am J Public Health.

[20] Krebs NF, Jacobson MS (2003). Prevention of pediatric overweight and obesity. Pediatrics.

[21] Centers for Disease Control and Prevention. 2000 CDC growth charts: United States.

[22] Story M, Holt K, Sofka D (2002). Bright futures in practice: nutrition.

[23] Kolagotla L, Adams W (2004). Ambulatory management of childhood obesity. Obes Res.

[24] Rhodes ET, Ebbeling CB, Meyers AF, Bayerl CT, Ooi WL, Bettencourt MF (2007). Pediatric obesity management: variation by specialty and awareness of guidelines. Clin Pediatr (Phila).

[25] Story MT, Neumark-Stzainer DR, Sherwood NE, Holt K, Sofka D, Trowbridge FL (2002). Management of child and adolescent obesity: attitudes, barriers, skills, and training needs among health care professionals. Pediatrics.

[26] Barlow SE, Trowbridge FL, Klish WJ, Dietz WH (2002). Treatment of child and adolescent obesity: reports from pediatricians, pediatric nurse practitioners, and registered dietitians. Pediatrics.

[27] Freemantle N, Harvey EL, Wolf F, Grimshaw JM, Grilli R, Bero LA (2000). Printed educational materials: effects on professional practice and health care outcomes. Cochrane Database Syst Rev.

[28] Oxman AD, Thomson MA, Davis DA, Haynes RB (1995). thetNo magic bullets: a systematic review of 102 trials of interventions to improve professional practiceitle. CMAJ.

[29] Davis DA, Taylor-Vaisey A (1997). Translating guidelines into practice. A systematic review of theoretic concepts, practical experience and research evidence in the adoption of clinical practice guidelines. CMAJ.

[30] McGuire WJ, Rice RE, Atkin CK (2001). theInput and output variables currently promising for constructing persuasive communicationstitle. Public communication campaigns.

